# Anti-Cancer Drug-Induced Lyell’s Syndrome: A Series of Two Patients

**DOI:** 10.3390/curroncol31110509

**Published:** 2024-11-04

**Authors:** Julie Coussirou, Magali Ravoire, Alma Stancu, Léa Vazquez

**Affiliations:** Institut du Cancer Avignon Provence, 250 Chemin de Baigne-Pieds, 84000 Avignon, France; j.coussirou@isc84.org (J.C.); m.ravoire@isc84.org (M.R.); a.stancu@isc84.org (A.S.)

**Keywords:** toxic epidermal necrolysis, Lyell’s syndrome, enfortumab vedotin, nivolumab, pharmacovigilance

## Abstract

Lyell’s syndrome or Toxic Epidermal Necrolysis (TEN) is a rare and life-threatening dermatological disease. Most commonly, this syndrome is drug-induced, and is a result of an immune-allergic reaction to medications. Anti-cancer drugs were not the most frequent groups of therapeutic agents related to Lyell’s syndrome, but the emergence of new therapeutic classes, particularly targeted therapy and immunotherapy, is changing current data. We present two cases of Lyell’s syndrome induced by anticancer drugs. (1) TEN in a man treated for metastatic urothelial carcinoma with Enfortumab Vedotin. (2) TEN in a man with metastatic melanoma treated with Nivolumab and Ipilimumab. Despite quick medical treatment and transfer to a severe burn unit, both patients died of TEN.

## 1. Introduction

Epidermal necrolysis, including Stevens–Johnson syndrome (SJS) and toxic epidermal necrolysis (TEN), is a rare but severe cutaneous disorder with high mortality and morbidity. Most of the time, this disorder is caused by an important drug immune-allergic reaction [1,2]. TEN, by covering more than 30% of the skin, is considered as the most life-threatening cutaneous reaction. Steven–Johnson Syndrome (SJS) differs from TEN in the inferior level of epidermal detachment (<10%). The annual incidence of SJS/TEN has been estimated to be from 1.4 to 2.26/million worldwide [3,4]. This drug-induced disease is most frequently caused by lactam antibiotics, antiepileptic drugs, allopurinol, and non-steroidal anti-inflammatory drugs. Chemotherapy, such as bendamustine, busulfan, chlorambucil, fludarabine, lomustine, and procarbazine, is also known to be related to severe skin disorders [5]. However, with targeted therapy development in cancer treatments and immunotherapy expansion, which are known for interfering with physiological mechanisms of peripheral immune tolerance, immune-related adverse events are increasing.

Enfortumab Vedotin (EV) is a promising treatment recently approved for locally advanced or metastatic urothelial carcinoma in the USA since December 2019. In France, patients can benefit from EV in a Compassionate Use Program, pending validation of the benefit–risk balance by the French Health Authorities. As a monoclonal antibody-microtubule inhibitor drug conjugate, EV is effective in cells that express Nectin-4, which is an adhesion immunoglobulin-like transmembrane molecule [6]. In clinical trials, the most common adverse events related to EV were rash, peripheral neuropathy, fatigue, alopecia, and nausea (grade 1–2 for 90%) [7]. Considering the important expression of Nectin-4 in epidermal keratinocytes, sweat glands, and hair follicles, mild cutaneous adverse reactions are predicted. There is a special warning about severe and fatal skin toxicities mentioned in the American and European summary of product characteristics of EV and a requirement for close skin monitoring. Likewise, as immune checkpoint inhibitors (ICIs), Nivolumab and Ipilimumab are known to cause mild cutaneous side effects (pruritus or rash), particularly in association (grade ≥ 3 for up to 3%); other immune-related adverse events are much more usual [8,9]. Nevertheless, with 1.5%, ICI-related deaths from dermatological toxic effects and fatal cutaneous adverse events are uncommon [10].

The objective of this report is to relate two patients who developed TEN syndrome after anti-cancer treatment.

## 2. Case Presentation

### 2.1. Case 1

A 71-year-old man with a medical history of diabetes and abdominal aortic aneurysm (treated with Metformin, Atorvastatin, acetylsalicylic acid, and hydrochlorothiazide) suffered from metastatic urothelial carcinoma with pulmonary metastases. In metastatic first-line treatment, the patient received four courses of Carboplatin-Gemcitabine followed by maintenance treatment with Avelumab. Already suffering from osteoarthritis and due to strong immuno-induced rheumatoid arthritis flare-ups, only three injections of Avelumab could be performed. Metastatic second-line treatment was started 4 months after stopping Avelumab, with Enfortumab Vedotin 1.25 mg/kg D1,8,15 on a 28-day cycle. Before initiation, the patient presented an ECOG Performance Status of 1 and grade 1 anorexia. Laboratory tests were within a normal range except for a known increase in creatinine (grade 2).

**Figure 1:**
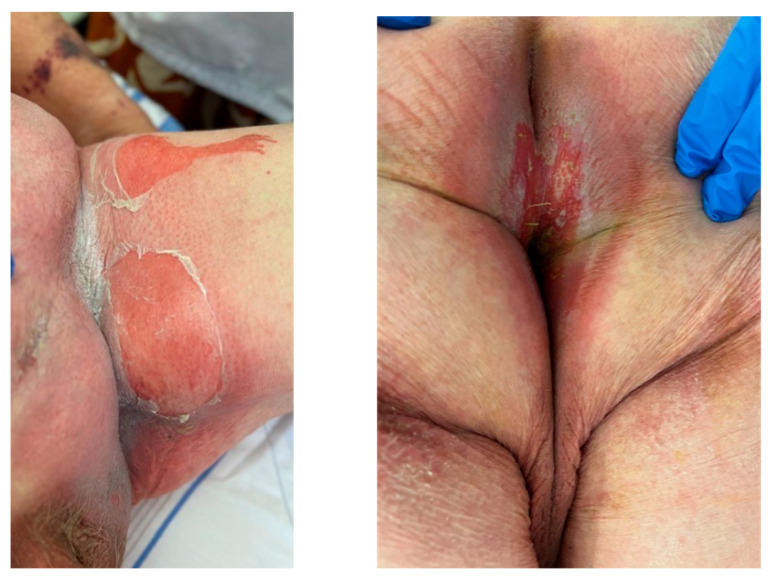
Skin reaction at admission, which was 7 days after his Enfortumab Vedotin Day 8 infusion.

The first week of his medication was well tolerated, with only grade 1–2 arthralgia. One week after his Day 8 infusion, he was admitted to hospital for bloody diarrhea and general epidermal detachment with large areas of exfoliation particularly involving bilateral inguinal folds, the entire anterior abdominal wall, left axillary region, and anal mucosa (Figure 1). The patient experienced severe pain and serious tiredness without fever or breathing difficulties. Laboratory tests revealed anemia (grade 1), leukopenia (grade 1), hypoalbuminemia (grade 2), and metabolic acidosis (high anion gap and loss of bicarbonates). Grade 3 hypercreatininemia was also compatible with renal failure. A skin biopsy was not performed. At hospitalization D1, his SCORTEN (severity-of-illness score for TEN) was ≥ 4 (serum urea and serum glucose were unknown). The patient was transferred to a specialized “severe burn” unit but died 6 days later despite treatments.

### 2.2. Case 2

**Figure 2:**
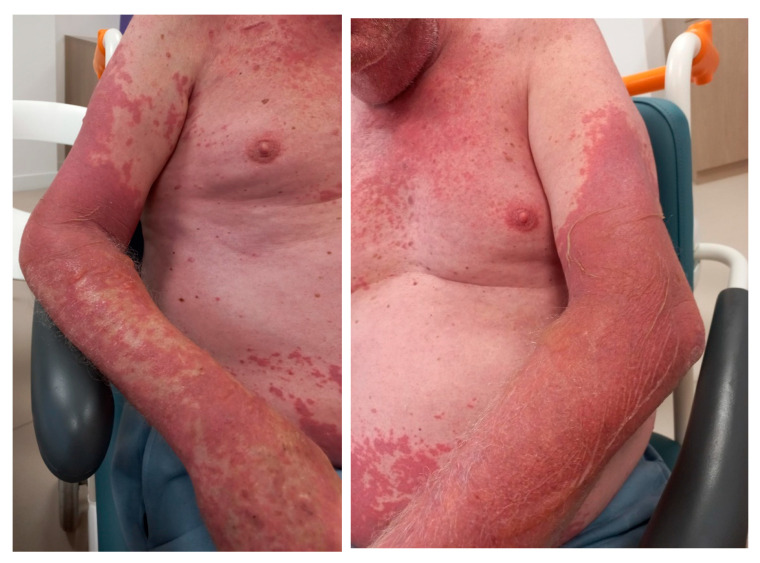
Skin reaction at admission, which was 13 days after Nivolumab infusion.

A 65-year-old man with a medical history of arterial hypertension (treated with angiotensin II receptor blockers) and depression (treated with Sertralin) was followed for recurrent melanoma with cerebral and pulmonary metastases associated with peritoneal carcinomatosis. The patient underwent stereotactic radiation therapy on cerebral metastases (3 × 11 Gy over 6 days) as a first-line treatment. Immunotherapy was scheduled three days later with Nivolumab 1 mg/kg plus Ipilimumab 3 mg/kg once every 3 weeks. On the first day of treatment, the patient developed a hypersensitivity reaction during Nivolumab perfusion with hyperthermia (39 °C), chills, and loss of consciousness. Samples for blood and urinary culture were performed, and the patient received antibiotics (Amoxicillin and Clavulanate 1 g). Antihistamines (Polaramine 5 mg) and a corticosteroid infusion (1.3 mg/kg) were effectively used for patient stabilization. Ipilimumab infusion was postponed to the next day and was well tolerated. However, on treatment initiation D10, an erythematous rash developed on 30% of his body with an oral involvement. A cutaneous adverse drug reaction due to antibiotics was suspected. No bacteria were identified in blood and urinary cultures; antibiotics were stopped. Local corticosteroids were added to ongoing antihistamines and oral corticosteroids (80 mg/j for brain metastases). Ten days later (D20), the patient was admitted to hospital for rashes, epidermal necrosis, and blisters on about 50% of his body (involving oral and conjunctival mucosa) associated with hyperthermia (39 °C), and tachycardia (120 bpm) (Figure 2). Laboratory tests revealed hypoalbuminemia (grade 1), but serum urea, serum glucose, and bicarbonate were within a normal range. Nivolumab-induced TEN was diagnosed with a SCORTEN of 4. The patient was transferred to a specialized “severe burn” unit. A skin biopsy was performed. An ophthalmologist confirmed bilateral eye damage and performed a subconjunctival injection of betamethasone. Parenteral support and analgesia were quickly started. At D29, 10 days later, because of multivisceral failure and acute respiratory distress syndrome, the patient died.

## 3. Discussion

SJS and TEN are immune-mediated cutaneous reactions characterized by epidermal necrosis involving more than 10% of the skin for SJS and more than 30% of the skin for TEN [11]. They are mostly drug-induced (80–95%) with several treatments commonly implicated, such as antibiotics (aminopenicillins, cephalosporins, and quinolones), allopurinol, non-steroidal anti-inflammatory drugs, and phenobarbital [3,12]. They develop within 1–2 weeks from the beginning of accountable drug therapy as observed for our two described patients. Already but rarely described as consequences of a chemotherapy regimen, in the age of targeted therapy development, this delayed-type drug-induced hypersensitivity reaction is increasingly reported in cancer patients [5,13,14,15,16,17]. The mortality rate of TEN ranges from 15% to 30% with several factors (advanced age, comorbidities, sepsis, and hematologic malignancies) associated with higher mortality risk [18,19].

The discovery of immune checkpoint proteins, acting as immune system suppressors, is a significant step in medical oncology and a leading approach in tumor immunotherapy. Given that checkpoint inhibitors interfere with the physiological mechanisms of peripheral immune tolerance and homeostasis, inflammatory (=immune-related) adverse events may affect any organ system, including the skin [20]. TEN induced by ICI therapy has rarely, but already, been reported [13]. While immune-mediated TEN induced non-specific erythema or dermatitis and a measles-like rash, common ICI immune-mediated adverse events include a grade 1–2 rash, which may lead to a delay in diagnosis [21]. The etiology of SJS and TEN has not been exactly elucidated, but CD8+ T cells seem to be implicated in the apoptosis of epidermal and mucosal epithelial cells. The specific mechanism remains unclear, but the over-activation of immune system theory, based on ICI proficiency to interfere with the maintenance of peripheral tolerance, is increasingly suspected by experts [22,23].

Enfortumab Vedotin (EV) is a novel, monoclonal antibody drug conjugate, approved for second or later-line therapy on patients with metastatic urothelial carcinoma [24]. Binding to Nectin-4 adhesion immunoglobulin-like transmembrane molecules expressed on tumor cells, EV induces cell apoptosis [25]. Considering that Nectin-4 is weakly to moderately expressed in normal tissues of the skin (cell–cell adhesion role), one of the most EV-related adverse events is skin reactions, with 43.9% of patients reporting rash; among these patients, 14.5% ≥ grade 3 [26]. In a pilot EV trial, topical corticosteroids were sufficient for complete rash resolution and 1 out of 125 patients developed SJS within 4 days of EV initiation. Although a TEN’s patient was treated successfully with systemic corticosteroids, this side effect was included in the safety warning of US prescribing information for EV. Specific recommendations such as treatment discontinuation upon the appearance of grade 2 lesions or rapid special burn unit transfer are urged. Indeed, TEN is associated with the highest mortality rate of all severe cutaneous adverse reactions because of all-organ involvement (erosion, necrosis, and severe dysfunction of the ocular, pulmonary, cardiovascular, gastrointestinal, and renal systems), leading to a potentially life-threatening process [7,27,28].

The severity-of-illness score for TEN (SCORTEN) is a mortality prognostication tool for epidermal necrolysis (SJS or TEN) [29]. Developed in 2000, this model identified seven equally weighted parameters as risk factors of death, which could predict the risk of mortality ranging from 3.2% for SCORTEN 0–1 to 90% for SCORTEN ≥ 5 [29,30]. The role of other biochemical markers has been investigated. The ratio of red cell distribution width to hemoglobin (RDW/Hb) was revealed to be efficient in predicting mortality risk [30] in TEN/SJS patients. Incorporating this value into the SCORTEN, the predictive accuracy improves. Indeed, Re-SCORTEN had a significantly better discrimination than SCORTEN alone (*p* = 0.02) [30]. Our two patients had a SCORTEN ≥ 4 at admission, i.e., an estimated mortality rate ≥ 58.3% [27] and a Re-SCORTEN ≥ 6, i.e., an estimated mortality rate ≥ 54.5% [28].

Despite its severity, TEN has no FDA-approved therapeutics in use. The immunopathogenesis, as a T-cell-mediated disease, involves CD8+ cell activation and other innate immune system cells for keratinocyte apoptosis induction. However, the cause of CD8+ activation remains unclear with two theories emerging: interaction with the immune system directly by pharmacologic interaction or indirectly by immunogenic molecule creation derived from drug digestion into metabolites that bind covalently to cellular peptides. Articular human leucocyte antigen allotypes seem to be involved in pathogenesis too [12]. This complicated pathogenesis makes the therapeutic strategy more difficult to standardize, especially since there is no curative treatment. An early diagnosis for rapid withdrawal of drugs associated with symptomatic and supportive treatment is the most relevant care to apply. Intensive skin care is critical and systemic glucocorticoids, immunoglobulin, or cyclosporine can help to reduce the skin reaction as well as plasmapheresis, but evidence for systemic treatment is still insufficient and controversial [31]. Unfortunately, a lack of information about the patients’ implemented treatment makes it inappropriate to discuss patient management.

The rarity and severity of SJS/TEN underscore the importance of accurate diagnostic criteria and effective treatments, which are currently lacking consensus [32].

## 4. Conclusions

Epidermal necrolysis (SJS/TEN), characterized by widespread sloughing of the skin and mucosal surfaces, is a rare but severe cutaneous drug-induced adverse reaction with a high mortality rate. A few cases have now been reported, but ICI-induced TEN is still considered rare (Table 1). Critical first steps in this grave adverse-event management are early diagnosis and rapid withdrawal. Therefore, TEN/SJS is a toxicity to keep in mind when treating cancer patients with recent targeted therapies, especially with a targeted therapy combination, which increase TEN/SJS risk.

## Figures and Tables

**Table 1:** Several recent cases of immunotherapy-induced Lyell’s syndrome.

Publication, Year	Age	Sex	Cancer Type	Immunotherapy	Time to Admission for TEN
[33] 2023	60	Man	HCC	Cadonilimab	29 and 8 days (2 injections)
[34] 2023	76	Man	Lung ADK	Sintilimab	9, 6, and 3 weeks (3 injections)
[35] 2023	75	Man	NSCLC and prostate cancer	Pembrolizumab	18, 15, 12, 9, 6, and 3 weeks (6 injections)
[36] 2023	75	Man	NSCLC	Pembrolizumab	15 days (1 injection)
[37] 2023	78	Man	Cholangio carcinoma	Sintilimab	19 days (1 injection)
[15] 2022	82	Man	Thymic carcinoma	Sintilimab	5 and 1 weeks (2 injections)
[38] 2022	71	Man	Bladder cancer	Enfortumab Vedotin	12 and 4 days (2 injections)
[39] 2022	45	Woman	Gastric cancer	Nivolumab	6, 4, and 2 weeks (3 injections)
Case 1	71	Man	Urothelial carcinoma	Enfortumab Vedotin	15 and 7 days (2 injections)
Case 2	65	Man	Melanoma	Nivolumab/Ipilumumab	20 days (1 injection)

## Data Availability

No new data were created.
